# Differences in addictive beliefs in people with psychostimulant addiction and in opioid addicts

**DOI:** 10.1192/j.eurpsy.2022.2117

**Published:** 2022-09-01

**Authors:** Y. Yan, E. Gutkevich

**Affiliations:** 1 National Research Tomsk State Universiy, Psychology Department, Tomsk, Russian Federation; 2 Tomsk national research medical center Russian Academy of Sciences, Department Of Endogenous Disorders, Tomsk, Russian Federation

**Keywords:** cognitive therapy, substanse use, Addiction, addictive beliefs

## Abstract

**Introduction:**

Today, a number of researchers consider the problem of addictive behavior as one of the most global problems for Kazakhstan and Russia. Some scientists consider CBT to be the most effective way to work with addictions. In our country there are no scientific works devoted to the study of addictive beliefs, so we decided to conduct such a study

**Objectives:**

The Objective of the study was to identify the characteristic addictive beliefs of drug addicts with different type of addiction: opioids and synthetic cathinones (designer drugs called “salts”, “bath salts”)

**Methods:**

Questionnaire of addictive beliefs by A, Beck, questionnaire of beliefs about cravings by A. Beck and F. Wright, clinical interview. Descriptive statistics and chi-square test were used for data processing

**Results:**

People with opioid addiction are more likely to believe that their lives will become more depressive if they stop using drugs (p= 0.0347); that drug use is the only way to cope with pain in their life (p= 0.0347) and that they cannot cope with anxiety without drugs (p=0.009). Respondents who use synthetic psychostimulants endorse to believe that addiction is not a problrm for them (p= 0.0358).

**Conclusions:**

Having categorized these beliefs in accordance with A. Beck’s classification, we came to the conclusion that “relief-oriented beliefs” are more typical for people who use opiates. The motive for use is often the desire to alleviate a negative emotional or physical state. For people using psychostimulants “salt”, “anticipatory beliefs” are more characteristic - the desire to experience euphoria and pleasant experiences

**Disclosure:**

No significant relationships.

